# Guide to Therapeutics and Materia Medica

**Published:** 1878-01

**Authors:** 


### BIBLIOGRAPHICAL.

Guide to Therapeutics and Materia Medica. By Robert Farquharson, M.D., Edin, F.R.C.P, London, Lecturer on Materia Medica, etc. Enlarged and adapted to the United States Pharmacopœia by Frank Woodbury, M.D., member of the Academy of Natural Sciences. Philadelphia, Henry C. Lea, 1877.

The novel arrangement of this work certainly gives it attraction which it would not otherwise possess. We confess that a feeling of more than the usual interest in ordinary compilations is elicited on this and other accounts. The author, unfortunately, thinks a purely physiological classification impracticable, because some remedies produce more than one action, thereby requiring, in such arrangement, the same article to be placed in different classes. On account of the confusion which the author thinks might thus arise, "it has been thought best to adopt the arrangement of Garrod and other popular text-books." It may be truly said, also, that authors and publishers do not wish to risk the popularity of new works by decided innovations in arrangement or anything else, even should they think them preferable.

A peculiar and attractive feature of the book consists in the arrangement of certain classes of remedies, so as, on the same page, to have a column each for the indications to be filled by the class, and the particular means used for the purpose. Twenty-two pages are thus occupied with the consideration of the following classes, in alphabetical order:

Antidotes, acids, anthelmintics, antipyretics, antiseptics, counter-irritants, diaphoretics, diuretics, emetics, emmenagogues, expectorants and purgatives.

The second part consists of over three hundred pages, occupied with individual "remedies comprised in the primary list of the United States Pharmacopœia," alphabetically arranged. The same arrangement of double column is also observed in this department of the work—one for the physiological action, the other for therapeutical results. This is a beautiful arrangement and strikes us as eminently practical, provided we have a particular remedy we desire to use, and wish to know what action may be expected from it. Convenience might have been better served, it is true, by having the class of actions tabulated, as in the first part of the work, and the articles arranged under such headings; for it is expected we always know what action is required before the remedy to produce it is sought. We think, therefore, while the plan is very nice, and affords a decided improvement on a majority of the works on therapeutics, that if all the remedies had been systematically noticed, as in the arrangement of the first part of the book, under the proper heads of classes, interest in it would have been increased greatly.

We are pleased to receive the work from the publisher, and would advise it as an addition, by all means, to the less useful works on this subject to be found in the libraries of physicians.

